# Supplementary material for: Statistical Analysis of Crystallization Database Links Protein Physico-Chemical Features with Crystallization Mechanisms
Source: PLoS One. 2014 Jul 2;9(7):e101123. doi: 10.1371/journal.pone.0101123 (PMC4079662; doi:10.1371/journal.pone.0101123)
Supplement: Materials S1 — List of the PDB ids of the proteins used in this study. (PDF) [file pone.0101123.s001.pdf]

List of the PDB ids of the proteins used in this study.

1l7y
1r57
1yez
1yh5
1zg2
2axo
2axp
2bdq
2bdv
2dlb
2es7
2es9
2euc
2ffg
2ffm
2fj6
2gf4
2gsw
2guj
2h3r
2h4o
2hh8
2i2l
2jn9
2js5
2jwy
2jxp
2JXT
2k1s
2K4N
2k50
2k52
2k57
2K5N
2K5W
2K75
2kck
2kcl
2KEN
2KF2
2kj6
2kpj
2krs
2krx

2kt9
2oyr
2p6y
2pgx
2ph1
2pif
2pih
2pkw
2q00
2qgg
2qgm
2qgp
2qgz
2qik
2qs9
2qti
2qzu
2r6z
2r76
2r7d
2ra2
2rad
2rb6
2rjb
3bdr
3bdu
3bf2
3bhp
3bid
3bij
3bu2
3c0d
3c37
3c3d
3c4s
3c96
3cer
3ceu
3cew
3cfu
3cnw
3cpk
3cq9
3cwi
3cwq
3d3n

3d3q
3d5n
3da1
3db9
3dc7
3dcd
3dcp
3dex
3dhn
3dkz
3dl3
3dm3
3dm4
3DMA
3dme
3DQP
3dr5
3DSM
3DTO
3E0E
3E0H
3ELI
3en2
3eqe
3ERJ
3esh
3esi
3evx
3ew7
3exz
3eyr
3f08
3f1t
3f1x
3f2i
3f3b
3f4k
3f4l
3fgb
3flh
3fnj
3g9q
3gek
3GGM
3GGN
3goc

3gvz
3gw4
3h2s
3h9n
3h9x
3HNR
3ht4
3hxj
3hxk
3i18
3I1H
3i23
3i3u
3igf
3ihk
3IPF
3jsr
3jvc
3jvn
3k1y
3k25
3K2T
3K2Y
3k7x
3K94
3KA5
3KA7
3kaw
3kb1
3kb4
3KBG
3KOJ
3KW6
3L8M
3LD7
3LKD
3lm8
3LMA
3lml
3lmm
3LYV
3lyw
3LYY
3E1E
3E5Z
3E8P
